# Plaque removal efficacy of a new toothbrush with a double-sided head and rotating handle—a pilot randomized control trial in acquired brain injury patients

**DOI:** 10.1007/s00784-023-05106-y

**Published:** 2023-06-30

**Authors:** Eliane García Mato, Lucía Sande López, Marcio Diniz Freitas, María Teresa Abeleira Pazos, Jacobo Limeres Posse, Pedro Diz Dios, Berta Rivas Mundiña

**Affiliations:** 1 Medical-Surgical Dentistry Research Group (OMEQUI), Health Research Institute of Santiago de Compostela (IDIS), University of Santiago de Compostela (USC), 15782 A Santiago de Compostela, Coruña Spain; 2 School of Medicine and Dentistry, C/ Entrerríos S/N, 15782 Santiago de Compostela, Spain

**Keywords:** Toothbrush, Dental plaque, Ergonomics, Acquired brain injury

## Abstract

**Objectives:**

To assess the efficacy of a new toothbrush (Balene) for the mechanical removal of dental plaque in patients with acquired brain injury.

**Material and methods:**

The study group consisted of 25 adults with acquired brain injury. The participants underwent 2 sessions of toothbrushing lasting 1 min, one with a conventional toothbrush and the other with the Balene toothbrush. This new double-headed toothbrush has 6 active sides, which allows for the simultaneous toothbrushing of both alveolar arches, with elastomer bristles angled at 45°, as well a handle that rotates up to 180°. Therefore, the user does not need to remove the toothbrush from the oral cavity during the toothbrushing process. Dental plaque accumulation was assessed using the simplified oral hygiene index of Greene and Vermillion.

**Results:**

The plaque index was significantly reduced both with the Balene toothbrush (*p* < 0.001) and with the conventional toothbrush (*p* < 0.001). The dental plaque removal efficacy was similar with the two toothbrushes. There were also no statistically significant differences in the removal of plaque with the Balene toothbrush between the autonomous and assisted toothbrushing modalities (*p* = 0.345).

**Conclusions:**

For patients with acquired brain injury, the Balene toothbrush was as effective as a conventional toothbrush, regardless of whether the toothbrushing modality was autonomous or assisted.

**Clinical relevance:**

The Balene® toothbrush’s efficacy in removing dental plaque is similar to that of conventional toothbrushes, both with the autonomous and assisted toothbrushing modality. Given its particular ergonomics, this toothbrush could be indicated for certain select patients with acquired brain injury (i.e., those whose degree of cooperation allows for toothbrushing, with a sufficient mouth opening, with no substantial abnormalities in the intermaxillary relationship, and with no significant edentulous sections).

## Introduction

A case of acquired brain injury (ABI) is defined as a patient who, after experiencing a brain injury of any origin, presents a prolonged motor, cognitive, emotional, behavioral, speech, and/or sensory deficiency, which reduces functionality and quality of life [1]. Although various etiological factors have been recognized, the main cause in Spain is stroke (80% of cases), followed by traumatic brain injury and, much less frequently, neurosurgical sequelae, cerebral anoxia, and central nervous system infections [1].

In general, individuals with ABI have poorer oral health than the general population, especially in terms of the prevalence of chronic generalized periodontitis, whose severity is determined by the frequency of toothbrushing [2]. Accordingly, the oral health-related quality of life (OHRQoL) of patients with ABI is poor starting the first weeks of hospitalization and is determined by the individual’s cognitive and motor skills and previous oral health [3]. The early introduction of oral care programs for patients with ABI has been shown to be effective in reducing the accumulation of bacterial plaque, improving their oral health and OHRQoL [4, 5].

Following a stroke (the most studied ABI condition in the literature), oral hygiene tends to be neglected due to neurological deficits, physical weakness, lack of coordination, cognitive dysfunction, and prioritization of other health needs [6]. As a result, these patients typically have poor oral health in terms of tooth loss, caries, and periodontal disease, in addition to a reduced likelihood of periodically visiting the dentist [7], and consequently have lower OHRQoL [8]. Deficient oral hygiene can also affect the recovery and rehabilitation of patients with stroke due to the risk of aspiration pneumonia, malnutrition, and social isolation [9].

Studies have confirmed that mechanical procedures for controlling plaque, and in particular toothbrushing, significantly decrease gingivitis and plaque accumulation [10]. For patients with ABI, however, toothbrushing is often compromised, given that motor difficulties entail less skill when handling the toothbrush, and cognitive impairment hinders patients’ understanding of the need for routine oral hygiene [11]. Therefore, the efficacy of various types of toothbrushes has been investigated in patients with motor and/or cognitive deficits [12]. These toothbrushes differ, among other characteristics, in the design of their head. The aim of this pilot study was to assess the efficacy in removing dental plaque of a new double-headed toothbrush (with 6 active sides) compared to a conventional adult single-headed toothbrush in patients with ABI, a group for whom there are very few scientifically validated recommendations on more appropriate toothbrushes. The null hypotheses were that the new double-headed toothbrush does not increase plaque reduction compared to the conventional toothbrush and that the toothbrushing modality (assisted vs. autonomous) has no impact on plaque reduction.

## Methods

A pilot study was designed as a randomized, controlled clinical trial and conducted between June and November 2022. A Balene® manual toothbrush was employed for half of the participants during the first appointment; as a control, a conventional manual toothbrush was employed during the second appointment (after a 2-week washout period). The order of the toothbrush implementation was reversed for the other half of the participants. To remove interparticipant variability from the intergroup comparisons, a crossover design was employed in which each participant served as their own control. To determine whether a participant should use the Balene® manual toothbrush or the conventional manual toothbrush during the first appointment, a system of randomization in balanced blocks (using numbered containers) was employed.

The study’s protocol was approved by the Ethics Committee of the University of Santiago de Compostela, Spain (registration number 2021/27). The participants, or their legal guardians (if necessary), completed and signed a specific informed consent for study participation. This article was prepared following the standards established for the presentation of information of trials (Consolidated Standards of Reporting Trials [CONSORT]) [13].

The inclusion criteria were an age greater than 18 years, confirmed diagnosis of ABI, and voluntary participation by signing an informed consent (either the patient or their legal representative). The following exclusion criteria were established: failure to provide a minimum of cooperation in allowing the toothbrushing to be performed, failure to achieve a mouth opening sufficient for performing the toothbrushing, not having at least one molar or premolar in each quadrant, and not having at least one maxillary incisor/canine and another mandibular incisor/canine.

Applying these criteria, 7 of the 32 users of the center were excluded, 3 for carrying a complete dental prosthesis, 3 for carrying a considerable fixed prosthesis (with more than 4 crowns), and 1 who was unable to sufficiently open their mouth. The pilot study’s participants consisted of 25 adults with ABI (12 men and 13 women between the ages of 41 and 74 years) who were members of the Alento Association for Individuals with ABI (Vigo, Spain). While 9 (36%) of the participants could perform autonomous toothbrushing, the remaining 16 (64%) required assistance to perform the toothbrushing. In 10 individuals (5 able to autonomously toothbrush and 5 requiring assistance), toothbrushing with the classical manual toothbrush was not performed due to a scheduling conflict with another activity in the center (6 participants), due to hospitalization (2 cases), or due to the expressed desire not to continue (2 cases). In this study, each participant used their preferred standard toothpaste (e.g., patients unable to spit out employed toothpaste without foaming agents). The supervision of the autonomous brushing and the performance of the assisted brushing were conducted by the regular caregivers of the Alento Association. With the objective of instructing the caregivers and patients with ABI on the toothbrushing technique with the Balene® toothbrush, the study’s authors conducted an information session lasting approximately 1 h, in which an informational video was projected and a direct demonstration conducted of the autonomous and assisted toothbrushing modalities.

**Fig. 1:**
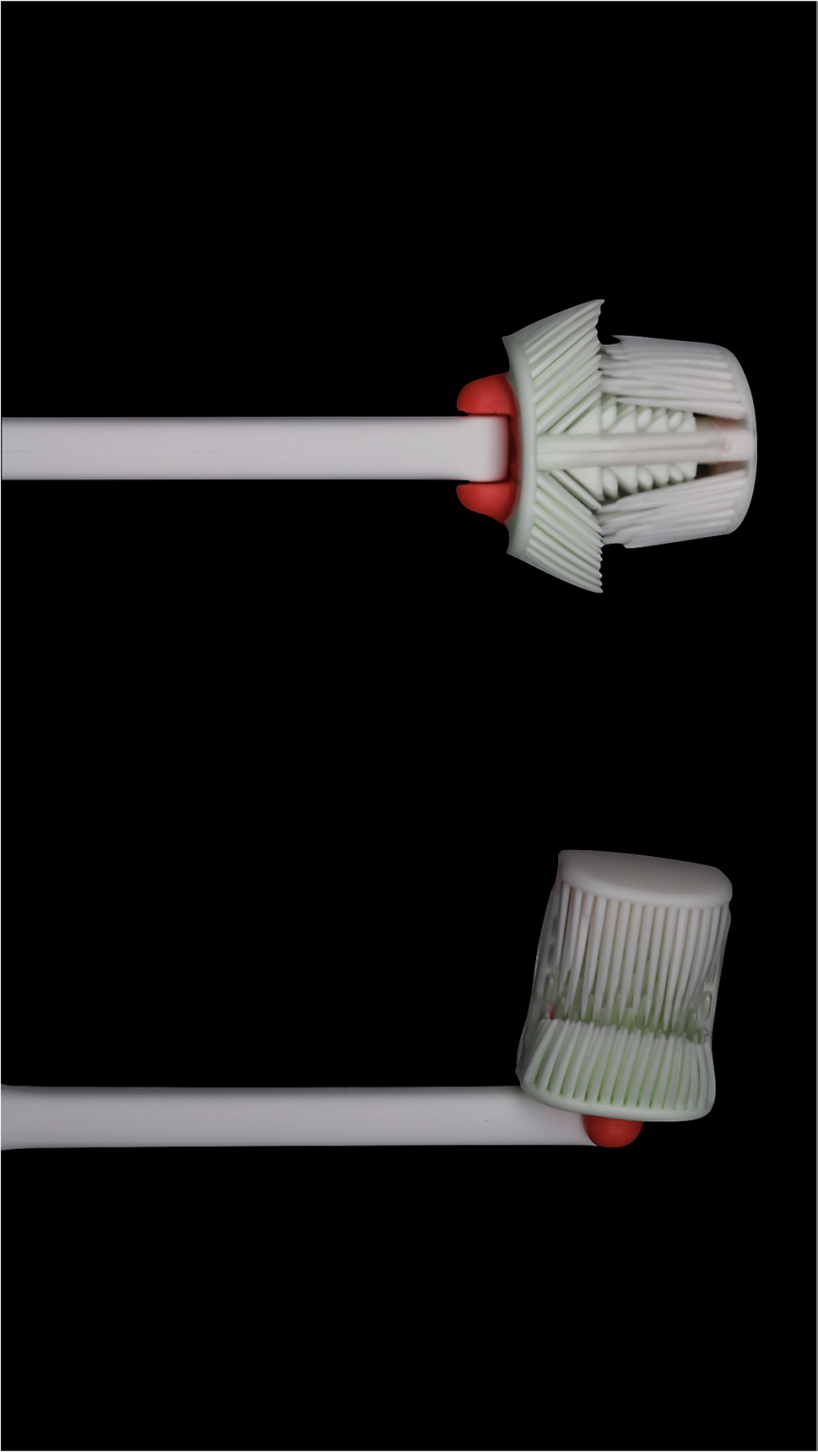
The new double-headed, 6-sided Balene® toothbrush with rotating handle

The manual Balene® toothbrush is characterized by two heads with 6 active sides, which allows for the simultaneous brushing of both the inner and outer sides of the upper and lower dental arcades. Its elastomer (thermoplastic polyurethane) bristles are angled at 45°, and its handle rotates up to 180°. The toothbrush does not therefore need to be removed from the oral cavity to change quadrant during toothbrushing (Fig. 1) (https://www.youtube.com/watch?v=CBqDgztnwwc). The reference toothbrush (control) was a conventional adult toothbrush with a single head and nylon bristles of medium hardness (Vitis®, Dentaid, Barcelona, Spain).

The participants’ teeth should not have been brushed in the 24 h prior to the appointment. Before starting each toothbrushing session, a plaque-disclosing agent (Plac control®, Dentaid, Barcelona, Spain) was applied with a mini-brush (Microbrush®, Dental Iberica, Mieres-Siero, Spain) to the dental surface of the teeth included in the simplified oral hygiene index of Greene and Vermillion [14]. The baseline quantity of plaque was recorded. Toothbrushing was then performed with the assigned toothbrush (Balene® or conventional) for 1 min (30 s for each quadrant). Once the toothbrushing was complete, the procedure for applying the plaque-disclosing agent was repeated, and the stained dental plaque values were once again recorded. The trial was conducted with blinding for the examiner (who waited in a nearby room during the toothbrushing sessions) and for the individual who performed the statistical analysis of the results.

This study considered independent variables the type of toothbrush (Balene® vs. conventional) and the toothbrushing modality (assisted vs. autonomous). The dependent variable was the quantification of the removed plaque. Two hypothesis tests were employed to perform the statistical analysis: the Wilcoxon signed-rank test and the Mann–Whitney U (Wilcoxon rank sum) test.

## Results

The range of the mean baseline plaque levels before toothbrushing with the Balene® toothbrush was 1.40–2.12. The range of the mean plaque accumulation after performing the toothbrushing with the Balene® toothbrush ranged from 0.48 in the mandibular central incisor to 0.88 in the maxillary first molar. After performing the toothbrushing, there was a statistically significant reduction in the total plaque accumulation (*p* < 0.001), in the accumulation on the incisors (*p* < 0.001), on the molars (*p* < 0.001), and on every individually analyzed tooth (*p* < 0.001 for all).

The range of the mean baseline plaque levels before toothbrushing with the conventional toothbrush was 1.53–2.40. After performing the toothbrushing with the conventional toothbrush, the range of the mean plaque accumulation ranged from 0.07 in the right maxillary central incisor and 0.87 in the right maxillary first molar. After performing the toothbrushing, there was a statistically significant reduction in total plaque accumulation (*p* < 0.001), in its accumulation on the incisors (*p* < 0.001), on the molars (*p* < 0.001), and on every individually analyzed tooth (ranging from *p* < 0.008 for the right maxillary first molar to *p* < 0.001 for the left maxillary first molar).

In terms of plaque reduction (pre- and post-toothbrushing), there were no statistically significant differences when comparing the results obtained with the Balene® toothbrush and those with the conventional toothbrush, except for that observed on the vestibular surface of the right maxillary central incisor, in which the conventional toothbrush was more effective (*p* = 0.021), this finding determining in turn the differences detected in all of the incisors (*p* = 0.028) (Table 1).

**Table 1:** Differences between the reduction in dental plaque levels achieved when performing toothbrushing with Balene® toothbrush and with the conventional manual toothbrush

Teeth involved	Balene® toothbrush	Conventional toothbrush	Difference	*P*
16 V	− 1.333	− 1.267	0.067	0.942
11 V	− 0.066	− 1.733	− 1.067	0.021
26 V	− 1.733	− 2.067	− 0.333	0.327
31 V	− 0.800	− 1.400	− 0.600	0.102
36 L	− 0.800	− 0.867	− 0.067	0.851
46 L	− 0.667	− 0.933	− 0.267	0.390
Posterior total	− 4.533	− 5.133	− 0.600	0.445
Anterior total	− 1.467	− 3.133	− 1.667	0.028
Total	− 6.000	− 8.267	− 2.267	0.055

*P*, statistical significance; 16 V, vestibular surface of the right maxillary first molar; 11 V, vestibular surface of the right maxillary central incisor; 26 V, vestibular surface of the left maxillary first molar; 31 V, vestibular surface of the left mandibular central incisor; 36L, lingual surface of the left mandibular first molar; 46L, lingual surface of the right mandibular first molar; Posterior total, total plaque accumulation on molars; Anterior total, total accumulation of plaque on incisors; Total, total plaque accumulation

When comparing the results obtained after autonomous toothbrushing and assisted toothbrushing using the Balene® toothbrush, statistically significant differences were not reached between the two toothbrushing modalities (*p* = 0.345).

## Discussion

This study was performed to evaluate the efficacy of a new double-headed toothbrush with rotating handle (Balene®) in removing dental plaque in patients with ABI. Although technological advances have helped improve the autonomy of individuals with disability and have facilitated their caregivers’ workload and that of health practitioners in providing dental care, attention to the oral health of patients with ABI is still very low [9]. To our knowledge, there have been no published studies on the efficacy of the Balene® toothbrush in patients with ABI.

This study showed that the Balene® toothbrush significantly removes dental plaque, a result confirmed by other authors who have indicated that toothbrushes with multiple heads reduce the accumulation of plaque, promoting the resolution of gingivitis and reaching difficult-to-access intraoral locations in healthy adults [15], older adults (> 65 years) [16], and children with intellectual disability [17].

No significant differences between the Balene® toothbrush and the conventional toothbrush in terms of plaque removal were observed in this trial, as has already been demonstrated in a group of systemically healthy volunteers [18]. In general, the literature supports the finding that toothbrushes with multiple heads are as effective as conventional toothbrushes with single heads [12, 15–17, 19]. Anecdotally, in a study conducted with patients younger than 18 years with intellectual disability who were able to perform autonomous toothbrushing, a toothbrush with 3 heads was more effective than a conventional toothbrush but less effective than an electric toothbrush with an oscillating-rotating head [20].

When using the Balene® toothbrush, there were no significant differences in plaque removal efficacy when comparing the modality of autonomous versus assisted toothbrushing. Although this study did not compare the efficacy of the Balene® toothbrush with that of the conventional toothbrush when considering the toothbrushing modality (due to sample size limitations), it has been suggested that, for patients with physical and/or cognitive disability, multi-head toothbrushes are especially indicated when total assistance is required to perform the toothbrushing [21]. A study on an institutionalized patient group with cerebral palsy who underwent assisted toothbrushing twice daily for 1 month also suggested that a toothbrush with 3 heads was more effective than a conventional toothbrush with a single head [22].

In systemically healthy individuals, the Balene® toothbrush was safer, and users rated its efficiency and efficacy as satisfactory [18]. One of the criteria for selecting the most appropriate oral hygiene device for patients with ABI is the personal preference of the patients themselves and that of their caregivers, based to their experience and expectations [9]. Caregivers who regularly perform assisted toothbrushing prefer multi-head toothbrushes over conventional toothbrushes [22]. Although this variable was not strictly assessed in this study, the healthcare practitioners involved considered that the Balene® toothbrush was more ergonomic and had advantages over a conventional toothbrush when they had to perform assisted toothbrushing in selected patients (e.g., without severe dental crowding, large edentulous sections, or dental malocclusions).

This pilot study is not exempt from certain limitations that require the results to be interpreted with caution. The sample size corresponds to that of a study group of advisability. Recruiting all participants of the same center has advantages in terms of supervision, diet, and routine but requires the assumption that the study’s conclusions cannot be extrapolated to other groups. Moreover, there is the possibility of a carry-over effect regarding the order in which the two toothbrushes were applied, given that this study did not analyze the carry-over effect, as well as the lack of attendance by certain participants to one of the appointments. With the oral hygiene index employed in this study [14], only 6 teeth were assessed; however, this index is typically used for individuals with disability because its implementation is very simple. It has been suggested that the use of a liquid dye to visualize the bacterial plaque can induce an overestimation of the existing plaque; however, a dye was employed in this study for effectiveness reasons because the measurements were not performed in a dental chair, and a number of the participants had difficulties keeping their mouth open without performing uncontrolled movements. Despite the fact that the recommendations for proper toothbrushing typically indicate a minimum duration of 2 min, the duration was set to 1 min for this study, based on an assisted toothbrushing test performed earlier with the center’s assistants before starting the study. Lastly, the new manual Balene® toothbrush is priced at approximately 15 euros, compared with 3–5 euros for a conventional manual toothbrush. However, the performance of the new thermoplastic polyurethane-bristle toothbrush compared with a conventional nylon-bristle toothbrush in terms of durability is still unknown.

## Conclusions

Despite the study’s limitations, the Balene® toothbrush was as safe and effective as a conventional toothbrush in removing dental plaque, regardless of whether the toothbrushing modality was autonomous or assisted. The Balene® toothbrush could therefore be indicated for certain selected patients with ABI (i.e., those whose degree of cooperation allows for toothbrushing, with a sufficient mouth opening, with no substantial abnormalities in the intermaxillary relationship, and with no significant edentulous sections).

## Data Availability

The data of this study are available from the corresponding author on request.
